# An anti-cancer surveillance by the interplay between interferon-beta and retinoblastoma protein RB1

**DOI:** 10.3389/fonc.2023.1173467

**Published:** 2023-04-27

**Authors:** Albert Qin

**Affiliations:** Medical Research & Clinical Operations, PharmaEssentia Corporation, Taipei, Taiwan

**Keywords:** tumorigenicity, cell cycle-based anti-cancer surveillance, tumor suppressor protein-mediated mechanism, interferon-beta (IFN-β), retinoblastoma protein RB1

## Abstract

Interferon-beta (IFN-β), an extracellular cytokine that initiates signaling pathways for gene regulation, has been demonstrated to function as a tumor suppressor protein through lentiviral gene transduction. In this article, I review the relevant previous works and propose a cell cycle-based, tumor suppressor protein-mediated mechanism of anti-cancer surveillance. IFN-β induces a tumor cell cycle alteration that leads to S phase accumulation, senescence entry, and a loss of tumorigenicity in solid tumor cells. IFN-β does not show a significant cell cycle effect in their normal counterparts. Retinoblastoma protein RB1, another tumor suppressor protein, tightly controls the cell cycle and differentiation of normal cells, preventing them from being significantly impacted by the IFN-β effect. The interplay between IFN-β and RB1 acts as a mechanism of cell cycle-based, tumor suppressor protein-mediated anti-cancer surveillance that can selectively suppress solid tumor or proliferating transformed cells from the loss of control leading to cancer. This mechanism has important implications for the treatment of solid tumors.

## Introduction

Cancer occurrence and growth involve the activation of oncogenes and loss or inactivation of tumor suppressor genes or proteins. During this process, events such as gene mutations, loss of tumor suppressor functions, and changes in intracellular and extracellular signaling networks accumulate and aggregate, leading to cell transformation or abnormal cell proliferation, cancer, and metastasis. The initial cell transformation or cancer cell formation may not necessarily be sustained to cause established cancer (1, 2). As cancer cells can often escape immune systems or immune surveillance may not always be adequate, it is not clear whether there are other mechanisms of surveillance and control against cancer development *in vivo*, and if so, how they exist.

### Type I interferons alpha and beta

Type I interferons (IFNs) including alpha (IFN-α) and beta (IFN-β), are extracellular cytokines. IFNs function by binding to receptors and activating the Janus tyrosine kinase signal transducer and activator of transcription (JAK-STAT) pathway or other signaling pathways to regulate various genes (3–6). IFN-β exists in a single form and is expressed in virtually all tissues and cell types. IFN-α has 13 subtypes and is noted to be produced in many types of cells including plasmacytoid dendritic and other immune cells (7–9). IFN-α and IFN-β bind to the type I IFN receptor (IFNAR), which is expressed on almost every cell type and is composed of the transmembrane subunits IFNAR1 and IFNAR2 (10–12). They induce similar anti-proliferative, immune-stimulatory, and anti-angiogenic activities (6, 12–15). The anti-proliferative effects include cell cycle inhibition and apoptosis (16–20).

**Figure 1:**
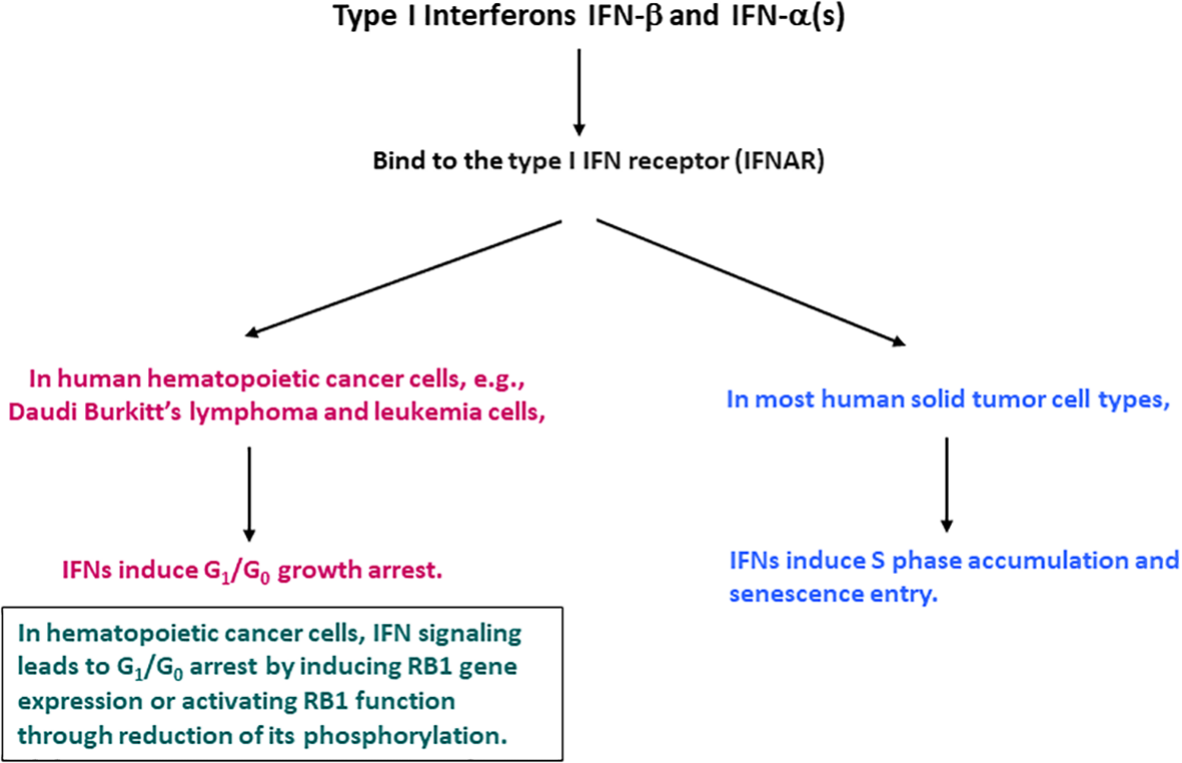
Cell cycle alterations induced by IFN-β and IFN-α in human hematopoietic cancer and solid tumor cells.

IFNs were reported to cause a cell cycle inhibition, including G_1_/G_0_ arrest, S phase prolongation, or both (16–18, 21). These effects appeared to be dependent on the cell type examined. In hematopoietic cancer cells, IFN-α and IFN-β cause G_1_/G_0_ cell cycle arrest. The G_1_/G_0_ arrest was the most characterized and considered to be the most common IFN effect (18, 22). Data mainly with IFN-α suggest that they induce the expression of the retinoblastoma protein (RB1) or activate its function by reducing its phosphorylation (23–30). RB1 is phosphorylated during the G1 phase and becomes hyper-phosphorylated at the G1 to S phase transition by cyclin-dependent kinases (CDKs) including cyclin D-CDK4/6 and cyclin E-CDK2 (31–38). IFN signaling could suppress CDK2, 4 and 6 activities or their regulatory cyclin subunits and induce gene expression of CDK inhibitors including p19^Ink4D^ and p21^WAF1/CIP1^, and phosphatase CDC25A (26–30). However, subsequent systematic analyses with various cell types indicated that in human solid tumor cells, the predominant IFN cell cycle effect might differ. IFN-β, and presumably IFN-α induce S phase accumulation and entry into senescence (19, 39). A diagram indicating the differential IFN-induced cell cycle alterations between human hematopoietic cancer and solid tumor cells is shown in **Figure 1**. Despite the notable similarity and redundancy in IFN-α and IFN-β activities, a recent study suggests that IFN-α subtypes may function mainly to complement, prolong, and amplify the IFN-β effects (40). Therefore, IFN-β represents a prototype of type I IFNs and meticulously mediates its activities potentially with the cooperative and mutually supplemental involvement of IFN-α subtypes.

### Anti-viral effect of IFNs and virus-induced carcinogenesis

Type I IFNs exhibit anti-viral activities (41, 42). The anti-viral activities can suppress virus-induced carcinogenesis. IFN signaling induces many antiviral effector proteins. These proteins can inhibit viral replication or augment the IFN-induced anti-viral response and include the anti-myxovirus-related (Mx) protein family with GTPase activity, 2′,5′-oligoadenylate synthetase 1, protein kinase R, and IFN-stimulated protein of 15 kDa (43). Viruses often antagonize IFN-induced antiviral responses to prevent their removal from cells. For example, hepatitis B virus downregulates *MxA* gene expression by its precore or core proteins (44) and SARS-CoV-2 virus inhibits endogenous IFN synthesis (45). In this respect, conjugation of polyethylene-glycol (PEG) on IFN molecule has significantly improved the pharmacokinetic profile of IFNs *in vivo* and allowed more convenient dose schemas than un-PEGylated recombinant IFNs. The PEGylation technology made IFN-based therapies possible with higher *in vivo* IFN exposures or longer half-lives that can endure the virus-mediated antagonizing effect. This improvement has led to clinically meaningful, therapeutic anti-viral responses (46–52).

Viruses, regardless of being genomic DNA or RNA-based, can impose cancer risk by introduction of viral oncogenes, activation of cellular oncogenes, or inactivation of tumor suppressor genes (53). This can disrupt normal growth and differentiation pathways leading to uncontrolled cellular proliferation and neoplastic transformation. Inflammation and other changes during viral infection can also elicit a virus-induced neoplastic process, potentially leading to cancer (54, 55). Therefore, anti-viral responses by IFNs may inhibit virus-induced tumorigenesis. In this respect, using an anti-viral regimen containing an anti-cancer component such as IFN-based therapy throughout the treatment course of onco-viral infections including hepatitis B or D may potentially minimize cancer occurrence (56).

### Immune surveillance

Immune surveillance is an important, well-characterized mechanism for detection and removal of cancer cells (57–59). Cancer progression occurs when cancer cells escape the immune surveillance as part of a process termed as cancer immunoediting (60, 61). IFNs play important roles in immune surveillance against cancer and the cancer immunoediting process during the interaction of cancer cells and the immune system (60, 61). IFN-α and IFN-β are strong immunostimulants (7–9). However, an immune activation by IFNs *per se* may not be directly linked to an antitumor effect in some cancer models. We explored the IFN-β-induced immune-based, antitumor effect using IFN-β gene therapy in tumor mouse models (62, 63). IFN-β gene therapy showed potent antitumor responses in various immune-deficient and -competent mouse models. The involvement of immune cells was subsequently defined by using depleting antibodies against immune cells (62, 63). The results indicated that the antitumor effect of IFN-β was dependent upon natural killer (NK) cells with a suspected macrophage involvement. Furthermore, IFN-β gene therapy significantly inhibited tumor growth and metastasis *via* cytotoxic CD8+ T-cells, even with the depletion of CD4+ T helper cells in immune-competent mice (63). In recent years, cancer cell-intrinsic signaling was found to significantly impact the tumor immune landscape (64). Animal modeling and research have provided further insights into our understanding of the effect of IFN-α and IFN-β signaling on the immunity against cancer. The interaction between type I IFN signaling and cellular oncoproteins such as c-MYC and KRAS were implicated in affecting the immune microenvironment including NK cell-mediated immunity (65–67), underscoring the importance of type I IFNs in immune surveillance against cancer.

## A mechanism of cell cycle-based, anti-cancer surveillance mediated by the interplay between IFN-β and RB1

An important and intriguing aspect of IFN-β, perhaps somewhat overlooked, is its direct tumor suppressor function, which is different from its general cytotoxic effects. In 2002, we reported our results on IFN-β gene delivery into tumor cells using a lentiviral vector, providing evidence that IFN-β can function as a direct or cancer cell-intrinsic, tumor suppressor protein (39). Abnormalities or deletions in chromosome 9p containing IFN-α, IFN-β, and other genes were previously observed to be frequent in cancer. However, IFN-β gene was not specifically identified to be relevant in cancer occurrence (68–70). We noted that human IFN-β induced overt apoptosis or cytotoxicity in human cancer cells when overexpressed by an adenovirus vector or in combination with chemotherapeutic agents (20, 62, 71). Therefore, we performed lentiviral vector-mediated IFN-β gene transduction with the initial aim of introducing the gene at a low copy number into human tumor cells and characterizing the IFN-β-induced cell cycle effect by separating it from cytotoxicity. Tumor cell clones stably expressing IFN-β were acquired after the gene transduction with the lentivirus. Despite stable IFN-β expression, the cells continuously divided and grew *in vitro*. All cell clones transduced by the IFN-β gene had a cell cycle alteration showing S phase accumulation and an entry into senescence. Importantly, all the cells lost their ability to form tumors *in vivo* when implanted back into animals. Therefore, IFN-β functioned as a tumor suppressor protein (39).

The cell cycle profile of the IFN-β-expressing clones was consistent with our earlier observations (19). Given the apparent lack of comprehensive understanding of the IFN-induced cell cycle effect in all cancer types, we used IFN-β in a systematic analysis of various types of human cancer cells and their normal cell counterparts (19). IFN-β did not significantly alter the cell cycle profiles of normal cells grown under normal conditions without growth factor stimulation (19). However, various solid tumor cell types altered their cycle, with more cells detected in the S phase. After further examination, the S phase accumulation, not the G_1_/G_0_ growth arrest that was observed in hematopoietic cancer cells, was found to be the cell cycle effect of IFN-β in various types of solid tumor and transformed cells (19). The S phase accumulation appears to be due to an inefficient S phase progression without a G2/M phase accumulation (19, 39), suggesting that an intra-S phase checkpoint is activated. Catastrophic cell death was observed in IFN-β expressing tumor cells (39), indicating that some cells might have moved to the next cell division without the S phase completion. In solid tumor and transformed cells that exhibited the IFN-β-induced S phase accumulation, IFN-β activated the JAK-STAT pathway, as revealed by tyrosine phosphorylation of STAT proteins and activation of DNA-binding complexes including the IFN-stimulated gene factor-3 (ISGF3) (19). Consistent with our work with lentiviral IFN-β gene transduction, we observed a portion of tumor cells exhibiting senescence entry after IFN-β treatment. In solid tumor and cells that showed slow S phase and senescent entry, there was a lack of functional RB1. RB1 function was lost due to gene mutations, inactivation by hyperphosphorylation, or binding of viral oncoproteins. The cell cycle alteration induced by IFN-β was not significant in non-transformed cell counterparts, nor in RB1^+/+^ tumor cells with an abundant presence of underphosphorylated RB1. In the latter, IFN-β signaling was clearly detected using electrophoretic mobility shift assays for active STAT transcriptional complexes including the transcription factor ISGF3 (19). Therefore, active RB1 in abundance prevented the RB1^+/+^ tumor cells from developing the IFN-β-induced cell cycle alterations despite IFN-β signaling.

RB1 is a tumor suppressor protein that provides key cell cycle regulation (72). It is phosphorylated at different phases of the cell cycle by cyclin D-CDK4/6 and cyclin E-CDK2 and becomes inactivated *via* hyperphosphorylation at the G_1_/S phase boundary possibly by cyclin E-CDK2, in a process regulated by CDK inhibitors (31–38). RB1 interacts with various cellular factors, including transcription factors E2F1-3 and histone deacetylase, to modulate gene expression and maintain normal cell cycle and differentiation (73–82). The loss of RB1, or disruption of its interaction with other cellular factors, is associated with a variety of human cancers (73). Our results indicate that in cells of solid tissue origin, the cell cycle regulatory machinery, hallmarked by functional RB1 interacting appropriately with cellular factors, is a determining factor for whether or not IFN-β induces cell cycle alterations (19).

**Figure 2:**
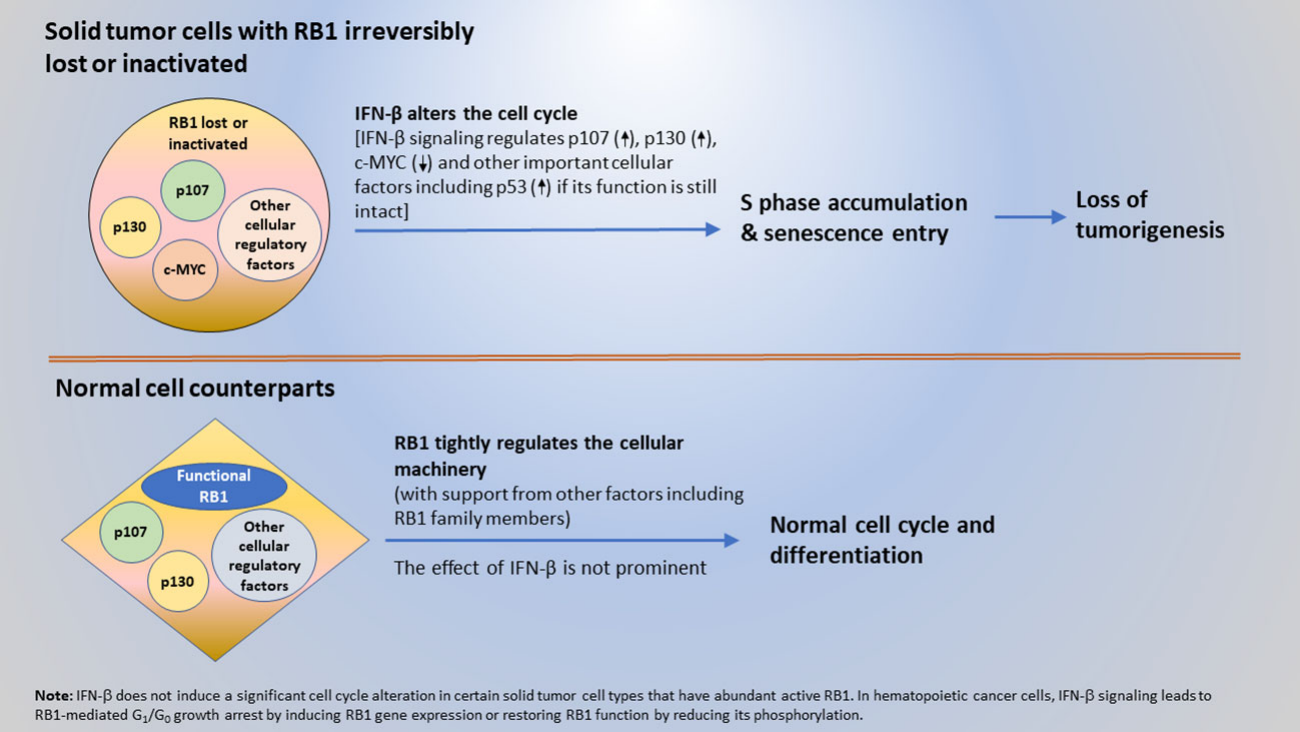
A mechanism of anti-cancer surveillance for selective suppression of tumor or transformed cells mediated by the interplay between tumor suppressor proteins IFN-β and RB1.

While the findings initially appeared perplexing, here it is proposed that a cancer cell-intrinsic, cell cycle-based mechanism of anti-cancer surveillance exists for the selective suppression of solid tumor and transformed cells. The extracellular and intracellular interplay between the tumor suppressor proteins, IFN-β and RB1, are central to this mechanism. Specifically, IFN-β signaling provides surveillance and removal of solid tumor and transformed cells by altering their cycle and causing more cells to accumulate in S phase and enter senescence. This effect leads to the loss of a tumorigenesis. Meanwhile, functional RB1 with its interacting cellular factors in normal cells to engage them in the normal cycle and promote differentiation, preventing them from being impacted by the IFN-β-induced cell cycle alterations. When RB1 function is lost due to gene mutation or protein inactivation, cells become susceptible to surveillance and inhibition by IFN-β. This tumor suppressor protein-mediated surveillance serves as a mechanism to selectively suppress solid tumor and transformed cells, including those that are occasionally derived from sporadic gene mutations and changes in epigenetic regulation. Furthermore, cell death in tumor cells that exhibited a very enlarged senescent cell phenotype was observed (39). Therefore, tumor cell senescence can ultimately lead to cell death and result in the removal of tumor cells. A schematic elucidating the mechanism of the tumor suppressor protein-mediated, cell cycle-based surveillance for the selective inhibition of solid tumor and transformed cells by IFN-β and RB1 is shown in **Figure 2**.

Most solid tumors have irreversibly lost RB1 function. Therefore, IFN-β signaling induces cell cycle alterations by interacting with cell cycle regulators other than RB1. However, the incidence of hematopoietic cancer varies. In Burkitt’s lymphoma (Daudi) and leukemia cells, loss of RB1 function appears to be reversible, and IFN-β signaling induces RB1 gene expression or restores RB1 function by reducing its phosphorylation (23–30). In these hematopoietic cancer cells, IFN-β exerts its tumor suppressor function *via* RB1-mediated G_1_/G_0_ growth arrest. The different IFN-β cell cycle effects illustrate that the cell cycle regulatory machinery differs between hematopoietic cancer and solid tumor cells.

## Discussion

IFN-β was revealed to possess a tumor suppressor protein function with lentiviral gene transduction (39). It alters the cell cycle of various solid tumor and transformed cell types by inducing S phase accumulation and senescence entry (19, 39). This article elucidates a new mechanism of anti-cancer surveillance mediated by the interplay between tumor suppressor proteins IFN-β and RB1. IFN-β in tissues maintains a presence for detecting tumor and transformed cells. Once a tumor cell is identified, IFN-β alters its cell cycle and causes an inefficient or slow S phase progression accompanied by senescence entry, rendering it no longer cancerous. RB1 interacts in this process in normal cells to engage them in their regular cycle and differentiation. The selective suppression of tumor and transformed cells without significantly affecting their normal counterparts, coordinated by IFN-β and RB1, is an important surveillance and control mechanism against cancer. As IFN-β is often significantly induced in response to viral infections that may impose a potential cancer risk, this cancer cell-intrinsic, cell cycle-based, tumor suppressor protein-mediated surveillance can suppress carcinogenesis or transformation at the cellular level during viral infections.

### Differential cell cycle regulatory machinery between hematopoietic cancer and solid tumor cells

In hematopoietic cancer cells, IFN-β signaling can directly restore RB1 function by increasing its gene expression or protein activation with reduction of its phosphorylation to induce an RB1-mediated G_1_/G_0_ growth arrest. As a result, there is a direct anti-cancer interaction between IFN-β signaling and RB1 function in the same cells. This differs from the cell cycle effects observed in various types of solid tumor cells, indicating an alternative landscape of the cell cycle regulatory machinery inside solid tumor cells compared with hematopoietic cancer cells. Indeed, the function of RB1 or its complexes that regulate the normal cell cycle are irreversibly lost or disrupted in most solid tumors. In addition, inactivation of the tumor suppressor protein p53 is very frequent in human cancer and could inhibit RB1 function as p53 can upregulate the CDK inhibitor p21^WAF1/CIP1^ from upstream (83, 84). Therefore, RB1-mediated growth arrest in G_1_/G_0_ by IFN-β does not occur in these solid tumor cells. Additional gene mutations or epigenetic changes present in solid tumor cells may help prevent proliferating cells from stopping at G_1_ and/or entering G_0_. Ultimately, these solid tumor cells undergo cell cycle alterations with an S phase accumulation due to a slow S phase progression possibly caused by an activation of intra-S phase checkpoint, and senescence entry in response to IFN-β signaling.

### Cellular proteins that IFN-β potentially agonizes or antagonizes for suppression of solid tumor cells

With slow S phase progression accompanied by senescence entry, not G_1_/G_0_ arrest, being the cell cycle effect of IFN-β in various types of solid tumor cells (19, 39), then how does IFN-β induce the effect in these cells in which RB1 function is irreversibly lost or inactivated? This article postulates that IFN-β does so in the solid tumor cells with irreversibly lost RB function by modulating the RB1 family members p107 and p130, and cellular factors, including c-MYC and other regulatory proteins. p107 and p130 can be associated with promoters in a similar manner as RB1 and cooperate with RB1 in cell cycle regulation, although with functional distinctions (85–88). In normal cells, p107 and p130 may complement or assist RB1 in regulating cell growth and differentiation. However, in tumor cells with irreversibly lost RB1 function, the involvement of p107 or p130 in cell cycle regulation becomes prominent. All RB1 family members are regulated by phosphorylation and bind to E2Fs with differential preferences to regulate gene expression (87, 89, 90). IFNs are suggested to be able to decrease all their phosphorylation (28). Notably different from RB1, which is phosphorylated as cells enter G_1_ from G_0_ and becomes hyperphosphorylated at the G_1_/S boundary, p107 is phosphorylated later in the cell cycle, in the late G_1_ and S phase onward (28, 91). Additionally, p107 was cloned as an RB1-related protein and is implicated in S phase regulation (92, 93). The expression of p107 in RB1-deficient osteosarcoma cells suppresses the progression of the S phase in addition to G_1_ (94). Therefore, one possibility is that in tumor cells with loss of RB1 function, IFN-β signaling reduces the phosphorylation of p107 to form p107/E2F or other p107 complexes to induce a p107-mediated suppression of the S phase progression. These complexes may suppress the genes promoting DNA synthesis and S phase progression (e.g., encoding cyclins, CDK1, DNA polymerase subunits, c-MYC, and B-MYB), resulting in the inhibition of S phase progression and more cells accumulating in the S phase. IFN-β also downregulates the growth-promoting gene *c-myc* in an RB1 family member-independent manner (95, 96), indicating that IFN-β activates different pathways to elicit its cell cycle effect. Newer data indicate that both p107 and p130 are involved in the senescence entry of tumor cells that have lost RB1 function (97–99). Downregulation of *c-myc* has been suggested to trigger tumor cell senescence (100–102). In addition, IFN-α and IFN-β signaling was shown to induce the transcription of the p53 gene by ISGF3 (103), suggesting that IFN-β signaling involves p53 in inducing the senescent entry of tumor cells retaining a functional p53 and an intact p53-responsive pathway. Moreover, IFN-α induces senescence-promoting CDK inhibitors including p19^Ink4D^ and p21^WAF1/CIP1^ (29, 30, 104, 105), which suggests that IFN-β signaling may also involve p19^Ink4D^ and p21^WAF1/CIP1^ in promoting senescence entry. Taken together, these data are consistent with our previous finding that in various types of solid tumor cells with the lost RB1 function, the prominent cell cycle effect of IFN-β is slow S phase progression and senescence entry accompanied by a loss of tumorigenicity. Additionally, a notion raised in this article is that IFN-β elicits its inhibitory effect on cell cycle and tumorigenicity in RB-defective solid tumor cells by inducing a p107-mediated suppression of S phase progression and regulating multiple cellular factors including p107, p130, c-MYC, and other important regulatory proteins.

### Clinical implications

The difference in the cell cycle machinery and effects induced by IFN-β between hematologic cancer and solid tumor cells has clinical implications. In hematologic cancers, IFN-β and IFN-α can induce a potent RB1-mediated G_1_/G_0_ growth arrest, implying that they are more sensitive to an IFN-based therapy. For solid tumors, however, adequate IFN-β or -α concentrations at the tumor sites to induce a significant cancer cell-intrinsic effect or immunity is a key for therapy success. Previously, clinical treatment for a broad range of solid tumors with IFN proteins, e.g., subcutaneous or intramuscular administration of un-PEGylated IFNs at millions of units/m^2^ multiple times per week, was generally not successful. This was likely due to an insufficient IFN level at the tumor site to induce an antitumor effect due to the rapid protein clearance after the treatment (20). Intratumor gene therapy with a replication-defective adenoviral vector encoding human IFN-β gene overcame the issue and led to a remarkable antitumor effect (20, 106). The recent approval of a non-replicating adenovirus encoding IFN-α 2b for high-risk Bacillus Calmette-Guérin-unresponsive non-muscle invasive bladder cancer by the US Food and Drug Administration (FDA) highlights the importance of sufficient local IFN levels in solid tumor treatment (107). Recent advances in PEGylation technologies yields IFN-based products with improved pharmacokinetic properties. The new advances may expand the opportunity to use IFN-based therapies in broader cancer indications including advanced metastasis. Ropeginterferon alfa-2b (also known as BESREMi) is a new-generation PEGylated IFN alfa-2b that is administered subcutaneously once every two or more weeks and currently in clinical development for several indications (108–112). In patients with polycythemia vera (PV), a myeloproliferative neoplasm, ropeginterferon alfa-2b provides clinically significant effects at the level of the patient’s bone marrow to selectively inhibit mutation-carrying malignant progenitor cells and increase the ratio of normal versus malignant cells (113–117). The FDA has approved ropeginterferon alfa-2b for the treatment of PV (118). Emerging data indicate that administration of ropeginterferon alfa-2b at a higher starting dose may lead to a quicker and greater level of complete hematological remission and reduction in the mutant variant allele frequency with manageable toxicities (119). Therefore, it is possible that a new generation IFN-based agent, such as ropeginterferon alfa-2b, may provide promising new treatment options for patients with a metastatic cancer at a favorable benefit-risk balance.

## Concluding remarks

Slowed S phase progression and senescence entry is the prominent cell cycle effect of IFN-β in various types of human solid tumor cells, not the G_1_/G_0_ growth arrest observed in hematopoietic cancer cells (19, 39). The effect is associated with a loss of tumorigenicity *in vivo* (39). This article elucidates a mechanism of cell cycle-based anti-cancer surveillance. The interplay between tumor suppressor proteins IFN-β and RB1 is central in this new mechanism of surveillance for selective suppression of tumor and transformed cells, while engaging their normal cell counterparts in regular cell cycle and differentiation. The IFN-β effect in various types of tumor cells with irreversibly lost RB1 function is possibly due to its signaling inducing a p107-mediated S phase inhibition and regulating p107, p130, c-MYC and other cellular factors including p53 if its function is still intact. For the future perspectives, the development of new therapeutic means to provide sufficient concentrations of IFN-β or α at tumor sites to achieve an antitumor effect is a key for the success of using an IFN-based therapy in cancer treatment.

## Author contributions

The author is the sole contributor of this work and has approved it for publication.
